# The impact of transformational leadership on the turnover intention of the new generation of knowledgeable employees: A moderated mediation model

**DOI:** 10.3389/fpsyg.2022.1090987

**Published:** 2023-01-26

**Authors:** Bin Xiong, Xiaoyan Wu, Qi Sui

**Affiliations:** School of Public Administration, Guangxi University, Nanning, China

**Keywords:** transformational leadership, the new generation of knowledgeable workers, turnover intention, person-organization fit, job embeddedness

## Abstract

The relationship between transformational leadership and employee behavior has been a popular topic in organizational research. However, while various factors have been identified for the influence of transformational leadership on employee behavior, researchers have so far failed to explore the impact of transformational leadership on the turnover propensity of the new generation of knowledge workers in terms of a specific orientation. Based on the social exchange theory, this study explored the influence of transformational leadership on the turnover intention of the new generation of knowledgeable employees, considering the mediating role of person–organization fit and the moderating role of job embeddedness. Through using SPSS 25.0, Amos 24.0, and PROCESS 3.3 to analyze the data of 326 workers, the results showed that transformational leadership has a negative predictive effect on the turnover intention of the new generation of knowledgeable employees. Person–organization fit plays a partial mediating role between transformational leadership and the turnover intention of the new generation of knowledgeable employees. The relationship between transformational leadership and person–organization fit is positively moderated by job embeddedness. Therefore, there is a moderated mediation model between transformational leadership and the new generation of knowledgeable employees. This research is a good reference and guide for management practices between transformational leadership and the new generation of knowledge workers.

## Introduction

The 14th 5-Year Plan of the Communist Party of China (CPC) clearly states that all organizations and units should innovate talent incentive methods and talent guarantee mechanisms, cultivate good people in an all-round way, and expand talent and innovation teams (The Xinhua News Agency, 2021). Currently, the post-80s and post-90s with higher education, professional knowledge and skills, and innovative vitality and potential have become important human resources of enterprises and public institutions. However, it is quite common for these new generations of knowledgeable workers to leave and frequently job-hop, which results in organizational management activities being faced with a lack of innovation and competitiveness, organizational performance being damaged, a divulge of organizational secrets, and the loss of important customers (Park and Shaw, 2013; An and Zhang, 2014; Hosseini et al., 2022). Therefore, reducing the turnover rate of the new generation of knowledgeable workers is the premise of the sustainable development of the organization. How to enhance the loyalty of the new generation of knowledgeable workers is an urgent problem to be solved by the organization at present.

Based on the theory of social exchange, transformational leadership, as a leadership style that emphasizes the common progress and mutual promotion of superiors and subordinates, is conducive to improving the innovative and enterprising consciousness of the new generation of knowledgeable workers and effectively strengthening their sense of ownership (Pu, 2015; Siangchokyoo et al., 2020; Abu-Rumman, 2021). Compared with other leadership styles, transformational leaders are good at using psychological authorization to meet the high-level needs of the new generation of employees in exchange for low-level turnover intentions of the new generation of employees (Zhang and Wang, 2017; Grošelj et al., 2020). Psychological authorization refers to the complex of empowering individual inner experience, including self-efficacy, self-determination, job meaning, and job impact (Shao et al., 2022). Although the academic circles are to explore the impact of transformational leadership on employee turnover behavior research, the new generation of knowledgeable workers as the research object discusses the impact of transformational leadership on their turnover intention as there is little research. On the one hand, transformational leadership can its quality exerts on Cenozoic knowledgeable employees to reduce their turnover intention remains to be further verification tested. On the other hand, previous research on transformational leadership mainly focused on the relationship between transformational leadership and the turnover intention of the new generation of knowledgeable workers at the internal level of the organization but did not combine the internal and external situations of the organization to conduct an in-depth analysis.

Previous studies showed that transformational leadership can create an inclusive and encouraging organizational atmosphere for employees to meet the needs of subordinates to be respected and improve the alignment of values between subordinates and the organization (Xu and Zhong, 2015; Xiong et al., 2017), and employees’ demands for change values and organizational aspects, such as the matching degree, can also be negative to predict employee turnover intention (Xu et al., 2015). In summary, transformational leadership and personal organization match affect the behavior of the staff, and between transformational leadership and personal organization match, there is also a certain logic. Therefore, the individual group match is in the transformational leadership of new generation ACTS as an intermediary role in the effect of knowledgeable employee turnover intention, and it remains to be further tested. In recent years, some studies tend to focus on employees’ interaction relations between and among organizations and families to explore employee behavior, such as family values match can reduce the pressure on employees’ work and improve employees’ work happiness (Liu et al., 2019), and leadership style as one of the direct factors affect organizational values; the relationship between Cenozoic group match of the knowledgeable employees level may be diverse situational factors. As a variable to study the degree of intimacy between employees and the organizational family network, influencing job embeddedness is often used to study employees’ dismission behavior (Xu et al., 2021), and the research on job embeddedness in the Chinese context is also expected to be related to employees’ leadership style. Organizational exchange relationships and other related variables were discussed together (Yang et al., 2010). However, there are few types of research that combine work embeddedness with transformational leadership style and employee turnover tendency.

To address these research gaps, exploring the relationship between transformational leadership and turnover tendency of the new generation of knowledge workers, based on the social exchange theory, we introduced individual–organization matching, a variable containing the interaction between organizations and employees, as the mediating variable to test whether it plays a mediating role in the influence of transformational leadership on turnover intention of the new generation of knowledge employees. At the same time, the variable of job embeddedness is introduced to consider the role of job embeddedness in the influencing mechanism of transformational leadership and turnover intention of the new generation of knowledge employees and to test whether job embeddedness plays a moderating role in the path of transformational leadership and individual–organization matching. Through in-depth analysis of the influence of transformational leadership on the turnover tendency of the new generation of knowledge employees, the theory of transformational leadership style and the development theory of knowledge workers are theoretically enriched, and in practice, it is beneficial to strengthen the close connection between leaders and the new generation of knowledge workers, so as to reduce the turnover rate of the new generation of knowledge workers.

## Theoretical basis and research assumptions

### Transformational leadership and turnover tendency of new generation of knowledge workers

Transformational leadership is a type of leadership proposed by American political sociologist Burns (1977) in his classic book on leadership in the 1980s. It is a leadership style in which leaders and employees enhance each other’s ethics and work motivation to a higher level. Based on the Chinese cultural context, Li and Shi (2005) believe that transformational leadership style can be interpreted from four characteristics: moral exemplarity, vision motivation, leadership charm, and personality concern. Many scholars based on the four traits from the aspects of psychological authorization, organizational identification, and cultural situation in China explored the relationship between transformational leadership and the new generation of employees and demonstrated the leadership style to the new-generation employees’ trust and follow, and degree of professionalism has a significant positive influence and effectively strengthens the close relationship between the superior and the subordinate (Li et al., 2015; Li and Mao, 2018; Liu and Li, 2018).

Referring to the definition of the new generation of knowledge workers by Wang and Yang (2017), in this study, the new generation of knowledge workers refers to the post-80s and post-90s who have received higher education, possess professional knowledge and skills, and have innovation vitality, autonomy, and potential. Turnover intention refers to a series of operations, such as looking for potential job opportunities and comparing potential positions with current ones, after employees have negative emotions such as job burnout and low job satisfaction in the process of work (Mobley, 1977). Due to the superior growth environment, Cenozoic knowledge-type employees tend to have higher levels of superiority and value pursuit, but their growth process is accompanied by early education, career choices, and peer comparison aspects of pressure, causing them to lack the courage to face setbacks and the ability to resist work pressure, leading to the high turnover (Ma, 2016).

According to the social exchange theory, there is a close exchange relationship between leaders and employees. For example, when employees feel appreciated and cared for by leaders, they tend to reward leaders with high emotional attachment and low turnover intention (Xu and Zhao, 2011). Combined with the personality traits of the new generation of knowledge workers, the reasons for their turnover thoughts can be summed up from the following three demand levels: (1) at the level of leader identification demand, the new generation of knowledge workers is eager to follow a leader with amiable character and high-level value pursuit. If the new generation of knowledge workers has low satisfaction with their leaders, it means that the employees are likely to have a high turnover intention (Ni, 2017). (2) In terms of results identification needs, the new generation of employees who prefer to be self-centered hopes that their work achievements can be recognized and praised by leaders and colleagues in a timely manner and even can be exchanged for actual remuneration or promotion. When the new generation of employees perceives that their work benefits are maximized and their work costs are minimized, their turnover intention will be negatively affected (Chen and Zheng, 2016). (3) The right to agree with the demand level and business ability of the new-generation employees desire to be a leader will give them certain privileges, making their judgment ability and creative thinking to get exercise, and outdated approval process and flexible way of working can inhibit their work motivation and job involvement. In summary, it can be said that leaders with different management styles have different levels of social interaction with their subordinates and have different levels of influence on their turnover plans. The new generation of knowledge workers with a high level of leader–member exchange will get more work resources and organizational support at work so as to improve their job satisfaction and organizational loyalty. On the contrary, the new generation of knowledge workers who have been ignored and excluded by leaders for a long time will greatly reduce their trust and recognition of leaders and even have the idea of leaving.

Based on the above elaboration and the characteristics of transformational leadership, this research believes that transformational leadership and the new generation of knowledge workers logically have a reciprocal relationship and emphasize that both sides progress together and promote each other. First of all, the good conduct of transformational leaders will subtly influence the new generation of knowledge workers so as to effectively enhance their close relationship with the new generation of knowledge workers in terms of values and behaviors. Second, the vision incentive characteristics of transformational leadership make it clear to the new generation of knowledge workers to convey the organization’s development prospects, fully show the organization’s development plan, and give the new generation of knowledge workers a certain degree of work autonomy to satisfy their desire for work discretion. Third, transformational leaders have the strong coping ability, the executive ability, and initiative and can timely solve practical problems for the organization and the new generation of knowledge workers. Through their work performance and personal benefits, they stimulate the new generation of knowledge workers to change their self-cognition and work engagement with positive images. Finally, in addition to providing support and encouragement at work, transformational leadership also provides material support and interpersonal care to solve the difficulties in the life of the new generation of knowledge workers so as to improve their sense of belonging and organizational dependence (Buil et al., 2019; Lin et al., 2020). It can be seen that transformational leadership can improve the new generation of knowledge workers’ dependence on themselves in many ways to reduce their turnover intention, which has a direct negative impact on the new generation of knowledge workers’ turnover decisions. In summary, this study proposes the following hypothesis:

H1: Transformational leadership negatively affects the turnover intention of the new generation of knowledge workers.

### The mediating role of individual–organization matching

Cable and DeRue (2002) divided individual–organization matching into three dimensions, value matching, need–supply matching, and competence requirement matching, and explained the differences in employee attitudes and behaviors with the interaction between individuals and organizations. Previous studies showed that the leader’s conduct style can not only effectively affect employee morale and job satisfaction but also significantly affect the organizational atmosphere and collective values. In combination with the personality traits of the new generation of knowledge workers, leaders do not pay attention to creating a fair internal atmosphere and a leadership style with high organizational support. This indicates that the values, needs, and other aspects of the new generation of knowledge workers are inconsistent with the collective values and material and spiritual supply presented by the organization. Under the condition of low matching degrees between individuals and organizations, the new generation of knowledge workers is likely to have a turnover tendency. For example, leaders ignore a series of behaviors that violate collective norms, such as group alliance and malicious internal competition, which will destroy the trust and follow of the new generation of employees, leading to the breakdown of the psychological contract between the new generation of employees and the organization, and further increase their turnover intention (Tao and Feng, 2020). Therefore, it is necessary to study whether individual–organization matching can be influenced by transformational leadership style, whether it can directly predict the turnover intention of the new generation of knowledge workers, and whether it can play a certain role in the path of transformational leadership affecting the turnover intention of the new generation of knowledge workers.

The relationship between transformational leadership and individual–organization matching shows that transformational leadership is good at using its value orientation to shape organizational climate. For example, transformational leadership pays attention to the shaping of learning organizations through values and creates an organizational atmosphere that encourages innovation and supports employees to realize self-value by enhancing collective effectiveness, promoting knowledge flow, and increasing creative output (Sun and Lei, 2018). Second, transformational leaders use their personalized care and leadership charm to create a highly supportive atmosphere. They pay attention to improving the level of interpersonal care in the organization from both material and spiritual aspects and motivate employees to improve their workability and personal quality with intellectual stimulation behavior so as to meet the complementarity between personal business ability and organizational position requirements. Based on the above analysis, transformational leadership can give full play to its value-oriented guiding role, the incentive role of material conditions, and psychological care and create an organizational atmosphere with high interpersonal care and team support so as to improve the matching degree between employees and the organization. Therefore, this study proposes the following hypothesis:

H2: Transformational leadership positively affects individual–organization matching.

The relationship between individual–organization matching and turnover intention of the new generation of knowledge workers is discussed. It has been found that the intention of the new generation of knowledge workers to stay and go is related to the degree to which their needs match the organization. From the perspective of three dimensions of individual–organization matching: value matching, need and supply matching, and ability and demand matching; first, the value deviation of individuals and organizations will lead to strong negative emotions in the new generation of knowledge workers, accompanied by a high turnover intention. The new generation of employees desires to be treated fairly by the organization in terms of profit distribution, job opportunities, and communication and interaction so as to encourage them to continue to stay in the collective and actively engage in work (Han, 2016). Second, according to social exchange theory, implicit mutual expectation between organization and employee, the organization provides the new generation of employees with expected material rewards and training opportunities in return for its mental activity. This kind of “high expectations–perception” reciprocal state can effectively reduce the new generation of employee turnover intention (Cheng and Lin, 2017). Finally, the new generation of employees needs to go through the process of socialization both in thought and action, and the matching of personal ability and job requirements requires constant running in with the organization in the process of interaction. Therefore, providing the new generation of employees with vocational training and training opportunities so that they can perceive the support and help provided by the organization for their long-term development is conducive to enhancing their willingness to stay (Li et al., 2020; Yu et al., 2021). Based on the above analysis, compared with considerable material remuneration, the new generation of knowledge workers with superior growth environments pays more attention to the positive value-oriented role of the organization in the distribution of benefits and interpersonal interaction. They are more eager to gain the respect and recognition of the organization and maintain a stable and mutually beneficial relationship with the organization and with the help of the organization through early career confusion to achieve socialization. If the new generation of knowledge workers cannot match the values of the organization, their expected demand deviates from the actual supply of the organization, and their career development cannot be helped and supported by the organization; they are very likely to quit. Therefore, this study proposes the following hypothesis:

H3: Individual–organization matching negatively affects the turnover intention of the new generation of knowledge workers.

To sum up, in the process of organization construction and operation, the leader is good at combining organization and the development of new generation knowledge staff, according to the organization culture and organization vision setting up a related system of value and salary, benefits, and consciously cultivating and corresponding organization atmosphere, the implementation of the corresponding human care, vocational training activities, to maintain the consistency between the new generation of knowledge workers and the organization in values, needs and supplies, capabilities and requirements so as to achieve low turnover intention. Therefore, this study holds that the influence of transformational leadership on the turnover intention of the new generation of knowledge workers can be transmitted as a mediating variable through individual–organization matching. Therefore, the following hypothesis is proposed:

H4: Individual–organization matching plays a mediating role in the impact of transformational leadership on the turnover intention of the new generation of knowledge workers.

### The moderating effect of job embedding

Job embedding was first proposed by American psychologist Mitchell et al. (2001) and used to study employee turnover behavior. It refers to the intimacy of the network formed between individuals and work-related situations inside and outside the organization. It mainly includes two forms: in-work embedding related to the organization and out-of-work embedding related to community and family. By combining these two embedding forms with attachment, matching and sacrifice, job embedding can be divided into six dimensions: organizational connection and community connection, organizational matching and community matching, organizational sacrifice and community sacrifice.

Yang et al. (2013) found through the interview that, under the influence of traditional Chinese culture, family has a very important influence on employees’ work and helps to increase employees’ social communication and improve their sense of integration and happiness. Similarly, before entering the organization, the new generation of knowledge workers always needs to consider their matching with the values and supplies of the organization, and there are also factors influencing the new generation of knowledge workers’ job choices in the community and family. For example, the pressure caused by commuting time and cost will be unconsciously added to the work, which will affect job satisfaction (Evans et al., 2002) and lead the new generation of knowledge workers to make other job choices. The leadership behavior style can not only improve the fit between the new generation of knowledge workers and the organization in all aspects from the organizational level but also meet the needs of employees in the community and family to a certain extent by means of workplace regulation and flexible working time (Nie and Xie, 2018). It can be seen that the extent to which transformational leadership can influence the matching of the new generation of knowledge workers with the organization is not only influenced by the in-job embedment related to the organization but also restricted by the out-job embedment related to the community and family. Therefore, to more comprehensively explore the level of influence of transformational leadership on individual–organizational matching, it is necessary to consider the moderating effect of job embedding.

Although there is a lack of literature research on transformational leadership and job embeddedness, some scholars have discussed the moderating effect of job embeddedness, which provides a logical basis for the research on job embeddedness and leadership style. As Wang et al. (2014) to explore the work embedded in the regulating role in the relationship between organizational commitment and innovation behavior, the high level of work embedded under the employees can feel more with the organization’s close ties with the conjunction, and the objective cognition can, in turn, strengthen the organization reliable on employees; Zhou et al. (2018) studied the moderating effect of job embedment on the relationship between psychological capital and employee engagement, suggesting that employee engagement at different levels of job embedment may be based on the rewards and benefits provided by the organization, or may be driven by psychological cognition. Therefore, transformational leadership, as one of the important factors, can positively affect organizational commitment and psychological capital (Liang and Li, 2016; Wang et al., 2017; Xenikou, 2017). Under the moderating effect of job embeddedness, the new generation of knowledge workers may adopt more favorable behaviors to match organizational values, supply, demand, and capabilities. In summary, this study proposes the following hypothesis:

H5: Job embeddings play a positive moderating role between transformational leadership and individual–organization matching. In other words, transformational leadership has a more significant positive impact on individual–organization matching at a high level of job embeddings.

Based on the above analysis and the previous hypothesis, this study concludes that job embedding moderates the mediating effect of individual–organization matching between transformational leadership and turnover intention of the new generation of knowledge workers. To be specific, transformational leadership will choose to improve the matching level between individuals and organizations based on the need to stabilize the turnover rate of the new generation of knowledge workers. However, a higher level of job embedment can make transformational leadership have a more positive impact on individual–organization matching. At this time, transformational leadership can reduce the turnover intention of the new generation of knowledge workers by improving the level of individual–organization matching. In conclusion, job embedding plays a moderating role in the relationship among transformational leadership, individual–organization matching, and the new generation of knowledge workers. In other words, the mediating role of individual–organization matching between transformational leadership and the new generation of knowledge workers is influenced by job embedding. Therefore, this study proposes a moderated mediation model hypothesis:

H6: Job embedment positively moderates the mediating effect of individual–organization matching between transformational leaders and the new generation of knowledge workers. In other words, at a high level of job embedment, individual–organization matching has a stronger mediating effect between transformational leaders and the new generation of knowledge workers.

**FIGURE 1:**
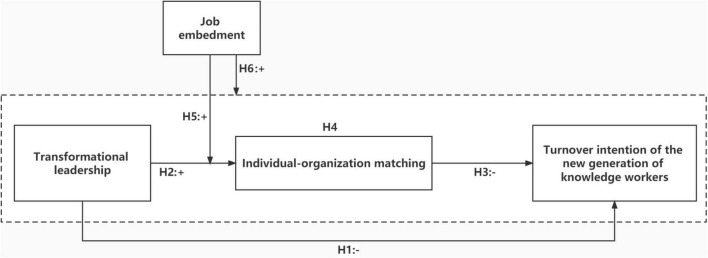
Theoretical model of this study.

According to the above research hypotheses, the theoretical model of this study is shown in Figure 1.

## Research design

### Research samples

This study mainly discusses the influence of transformational leadership on the turnover intention of the new generation of knowledge workers. The survey scope is mainly in Guangxi, and the knowledge workers born after 1980 are the sample. In June 2021 and September 2021, 400 questionnaires were distributed and 357 were recovered. In total, 31 invalid questionnaires that were incomplete and did not meet the age requirements were deleted. A total of 326 valid questionnaires were obtained with an effective recovery rate of 81.5%.

The basic information of the respondents is shown in Table 1, including 152 men, accounting for 46.6%, and 174 women, accounting for 53.4%. There were 167 under 25 years old (51.2%), 114 between 25 and 30 years old (35%), 38 between 30 and 35 years old (11.7%), and 7 between 35 and 40 years old (2.1%). Most of the respondents had bachelor’s degrees, accounting for 77%, and 75 had master’s degrees or above, accounting for 23%. Unmarried people accounted for the majority (77.3%) of the total sample; married people without children accounted for 8%; married people with children accounted for 14.4%; and divorced people with children accounted for 0.3%. In addition, among the new generation of knowledge workers surveyed, state-owned enterprises account for 27.6% of the total sample, government institutions account for 25.2%, private enterprises account for 32.2%, foreign enterprises account for 3.4%, Sino-foreign joint ventures account for 11.3%, and other units account for 0.3%; most of the respondents are ordinary employees, accounting for 69.3% of the total sample, ordinary managers account for 21.8%, middle managers account for 6.4%, and top managers account for 2.5%. At the same time, respondents almost completely filled in the working years and other relevant information.

**TABLE 1:** Basic information of respondents (*N* = 326).

Project	Category	Number	Percentage (%)	Project	Category	Number	Percentage (%)
Gender	Man	152	46.6	Unit properties			
	Woman	174	53.4		State-owned enterprise	90	27.6
					Government organs	82	25.2
Age	<25	167	51.2		The private enterprise	105	32.2
	25–30	114	35.0		The foreign capital enterprise	11	3.4
	30–35	38	11.7		Sino-foreign joint venture	37	11.3
	35–40	7	2.1		Else	1	0.3
Final academic degree	Bachelor	251	77				
	Master degree or above	75	23	Time in job	<1	101	31.0
					1–3	128	39.3
Marital status	Unmarried	252	77.3		3–6	65	19.9
	Married without children	26	8.0		6–10	21	6.4
	Married with children	47	14.4		>10	11	3.4
	Divorce with children	1	0.3				
				Years of work in the unit	<1	114	35.0
Position	Ordinary worker	226	69.3		1–3	125	38.3
	General manager	71	21.8		3–6	62	19.0
	Middle-level manager	21	6.4		6–10	16	4.9
	Top management level	8	2.5		>10	9	2.8

### Variable measurement

The measurement variables of this study include transformational leadership, the turnover intention of the new generation of knowledge workers, personal–organization matching, and job embedment. A maturity scale commonly used at home and abroad is adopted, and appropriate modifications are made based on expert opinions and research needs. The scale used is a 5-point Likert scale with a score of 1–5, representing “strongly disagree” to “strongly agree,” respectively. At the same time, to avoid the influence of demographic variables on the statistical results, this study takes basic information such as age and gender as control variables.

(1) Transformational leadership. The scale developed by Li and Shi (2005) is used to measure transformational leadership, which consists of 11 items in four dimensions, namely, moral model, vision motivation, leadership charm, and personalized care. Among them, the moral model contains three items, such as “your leadership can endure hardship first, enjoy after” and so on; vision motivation includes three items, such as “leaders often analyze the impact of their work on the overall goals of the unit and department together with employees”; leadership charisma includes three items, such as “your leader can constantly learn to enrich and improve themselves”; personality care includes two questions, such as “your leader is willing to help employees solve problems in life and family.” The Cronbach’s α coefficient of the scale was 0.949.

(2) Turnover tendency of the new generation of knowledge workers. The turnover intention scale developed by Mobley et al. (1978) is used to measure the turnover intention of the new generation of knowledge workers, with a total of 4 items, such as “you are very likely to leave the current unit in the short term” and so on. The Cronbach’s α coefficient of the scale was 0.905.

(3) Individual–organization matching. The measurement scale of Cable and DeRue (2002) was used to measure individual–tissue matching. The scale contains three dimensions: value matching, demand–supply matching, and demand–ability matching, with a total of nine items. Among them, the value matching dimension contains three items, such as “your values can match the values and culture of the organization”; the demand–supply matching dimension contains three items, including “your current job is exactly the job you are looking for, and it provides you with the material and spiritual resources you need.” Requirements–the competency matching dimension contains three items, such as “the skills you have are well matched to the job requirements.” The Cronbach’s α coefficient of the scale was 0.919.

(4) Job embedding. To satisfy the research in the Chinese context, the job embeddedness scale of Liang (2005) was used. The scale was divided into six dimensions of organizational bonding and community bonding, organizational matching and community matching, and organizational sacrifice and community sacrifice with a total of 15 items. Among them, the organizational connection includes two items, including “there are many colleagues who can be highly relied on in work”; the organizational match includes four questions, including “your current job makes the best use of your skills and talents.” Organizational sacrifice includes three items such as “you have nothing to lose if you leave the organization.” The community connection includes two items such as “you have a parent living in the area, or a caring family member or close friend living in the area.” Community matching includes two items, such as “do you like where you live now”; community sacrifice includes two questions: it is difficult to leave the community you live in. The Cronbach’s α coefficient of the scale was 0.901.

## Research results

### Common method deviation test

This study adopted a cross-sectional study. All questionnaire data were obtained at one time by the self-filling method of the respondents, so there may be a common method bias problem. According to the suggestion of Zhou and Long (2004), the control method of common method deviation can start from procedural control and statistical control. In terms of procedural control, this study used an electronic questionnaire and a paper questionnaire, a questionnaire anonymity method, advanced description of research purpose, pretest to improve scale items, and other methods to reduce the common deviation. In the statistical control, the Harman’s single-factor test is used to test the common method deviation. Through the unrotated principal component factor analysis, it was found that a total of seven factors were extracted from the principal component analysis, and the cumulative explanation of the seven factors was 68.679%, indicating that all the variation could not be explained by a single factor. Moreover, the variation explained by the first factor was 38.9%, which did not exceed the critical value of 40%, indicating that there was no serious common method bias in the data collected in this study.

### Confirmatory factor analysis

Amos 24.0 was used to test the construct validity of four measurement scales: transformational leadership, turnover intention, person–organization matching, and job embedding. According to the fitting index criterion of Hu and Bentler (1999) (X2/DF < 3, RMSEA < 0.08, CFI, AVE, CR, NFI, RFI, IFI, TLI fitting index > 0.9), Table 2 shows the relevant fitting index of each measurement scale, and Table 3 shows the HTMT result of each measurement scale. All measurement scales meet the reference standard. It indicates that the model in this study has a good fitting degree and good validity.

**TABLE 2:** Confirmatory factor analysis results (*N* = 326).

	X^2^/df	RMSEA	CFI	NFI	RFI	IFI	TLI	AVE	CR
Transformational leadership	2.895	0.076	0.977	0.965	0.946	0.977	0.964	0.628	0.936
Turnover intention	2.393	0.065	0.997	0.994	0.983	0.997	0.990	0.707	0.893
Individual–organization matching	2.627	0.071	0.984	0.974	0.961	0.984	0.976	0.642	0.773
Work embedded	1.928	0.053	0.968	0.936	0.911	0.968	0.955	0.511	0.735

**TABLE 3:** Heterotrait-monotrait ratio result.

	Transformational leadership	Personal–organization matching	Work embedding	Knowledge-based employees	The turnover tendency of the new generation of knowledge-based employees	Age	Gender
Transformational leadership	-						
Personal–organization matching	0.583	-					
Work embedding	0.465	0.515	-				
Knowledge-based employees	0.654	0.634	0.591	-			
The turnover tendency of the new generation of knowledge-based employees	-0.428	-0.424	-0.298	-0.513	-		
Age	-0.047	0.012	0.080	0.201	-0.080	-	
Gender	0.748	0.761	0.604	0.589	-0.385	0.165	

### Variable description and correlation analysis

In this study, Pearson’s correlation coefficient was used to test the relationship among transformational leadership, turnover intention of the new generation of knowledge workers, individual–organization matching, and job embeddedness, and gender and age are added as two control variables. As shown in Table 4, transformational leadership is significantly negatively correlated with the turnover intention of new-generation knowledge workers (*r* = −0.390, *P* < 0.01) and positively correlated with individual–organization matching (*r* = 0.593, *P* < 0.01). Meanwhile, there was a significant negative correlation between the personal–organization matching and the turnover intention of the new generation of knowledge workers (*r* = −0.385, *P* < 0.01). The results showed that H1, H2, and H3 have been verified, which provides preliminary support for the research hypothesis in this study.

**TABLE 4:** Descriptive statistics and correlation analysis (*N* = 326).

	*M*	SD	1	2	3	4	5	6
Gender	1.53	0.50	1					
Age	1.65	0.77	0.003	1				
Transformational leadership	3.32	0.70	0.099	-0.049	1	-0.390**	0.593**	0.607**
The turnover tendency of the new generation of Knowledge-based employees	3.56	0.86	0.013	-0.074	-0.390**	1	-0.385**	-0.420**
Personal–organization matching	3.32	0.67	-0.032	0.072	0.593**	-0.385**	1	0.645**
Work embedding	3.22	0.57	0.008	0.076	0.607**	-0.420**	0.645**	1

**Denotes *P* < 0.01.

### Moderated mediation model testing

Under the condition that the control variables are gender and age, this study first uses Model 4 in the SPSS macro program PROCESS compiled by Hayes (Model 4 is a simple mediation model) to test the mediating effect of individual–organization matching between transformational leadership and the turnover intention of the new generation of knowledge workers. As shown in Table 5, transformational leadership has a significant negative predictive effect on the turnover intention of the new generation of knowledge workers (β = −0.49, *t* = −7.79, *P* < 0.01), and the negative predictive effect of transformational leadership on the turnover intention of the new generation of knowledge workers is still significant (β = −0.33, *P* < 0.01) when the mediating variable individual–organization matching is included (*T* = −4.18, *P* < 0.01). Transformational leadership had a significant positive predictive effect on the personal–organization matching (β = 0.58, *t* = 13.64, *P* < 0.01) and a significant negative predictive effect on the turnover intention of the new generation of knowledge workers (β = −0.29, *t* = −3.52, *P* < 0.01).

**TABLE 5:** Test of the mediating effect of person–organization matching.

Regression equation (*N* = 326)		Fitting index			Significance of coefficient	
Outcome variable	Predictor variable	*R*	*R* ^2^	*F*	β	*t*
Turnover intention		0.41	0.17	22.31**		
	Gender				0.09	1.04
	Age				-1.04	-1.83**
	Transformational leadership				-0.49	-7.79**
Individual–organization matching		0.60	0.36	60.76**		
	Gender				-0.12	-2.09*
	Age				0.09	2.29*
	Transformational leadership				0.58	13.64**
Turnover intention		0.46	0.21	21.06**		
	Gender				0.06	0.64
	Age				-0.08	-1.40
	Individual–organization matching				-0.29	-3.52**
	Transformational leadership				-0.33	-4.18**

*Denotes *P* < 0.05 and **denotes *P* < 0.01.

As shown in Table 6, the upper and lower limits of the bootstrap 95% confidence interval of the direct effect of transformational leadership on the turnover intention of the new generation of knowledge workers are (−0.18, −0.47), respectively. The upper and lower limits of the bootstrap 95% confidence interval of the mediating effect of individual–organization matching between transformational leadership and turnover intention of new-generation knowledge workers are (−0.08) and (−0.29), respectively, excluding 0. The results showed that transformational leadership can not only directly predict the turnover intention of the new generation of knowledge workers, but also predict the turnover intention through the mediating effect of individual–organization matching, which plays a part of the mediating effect, and the direct effect and mediating effect account for 65.27 and 34.75% of the total effect, respectively. The results show that H1, H2, and H3 are further verified, and H4 is preliminarily supported.

**TABLE 6:** Breakdown table of total effect, direct effect, and mediating effect.

	Effect value	BootSE standard error	BootLLCI floor	BootLLCI ceiling	Relative effect value
Mediating effect	-0.18	0.05	-0.29	-0.08	34.75%
Direct effect	-0.33	0.08	-0.47	-0.18	65.27%
Total effect	-0.51	0.79	-0.48	-0.17	

Then, Model 7 in the SPSS macro program PROCESS compiled by Hayes (Model 7 assumes that the first half of the mediation model is moderated, which is the same as the theoretical model in this study) was used to test the moderated mediation model after adding the control variables, gender and age. As shown in Table 7, when job embedding was added to the model, the product term of transformational leadership and job embedding had a significant predictive effect on individual–organization matching (β = 0.15, *t* = 2.89, *P* < 0.01), indicating that job embedding could play a moderating role between transformational leadership and individual–organization matching.

**TABLE 7:** Test of moderated mediation model.

Regression equation (*N* = 326)		Fitting index			Significance of coefficient	
Outcome variable	Predictor variable	*R*	*R* ^2^	*F*	β	*t*
Individual–organization matching		0.70	0.49	62.45**		
	Gender				-0.09	-1.75
	Age				0.03	0.79
	Transformational leadership				0.33	6.81**
	Job embeddedness				0.49	8.20**
	Transformational leadership × job embeddedness				0.15	2.89**
Turnover intention		0.46	0.21	21.06**		
	Gender				0.06	0.70
	Age				-0.09	-1.57
	Transformational leadership				-0.33	-4.28**
	Individual–organization matching				-0.31	-3.81**

**Denotes *P* < 0.01.

To observe the effect of job embedding at different levels, the mean plus one standard deviation (M + 1 SD) was set as high job embedding, and the mean minus one standard deviation (M-1 SD) was set as low job embedding. Then, the bootstrap method was used to test the mediating effect value and 95% confidence interval results between transformational leadership and the turnover intention of the new generation of knowledge workers. As shown in Table 8, in the influence path of transformational leadership on individual–organization matching, the mediating effect of individual–organization matching is significant at the low level of job embedding, the effect value is −0.07, and the 95% confidence interval is (−0.02, −0.14), excluding 0. At a high job embedment level, the mediating effect of person–organization matching was also significant, with an effect value of −0.12 and 95% confidence interval of (−0.05, −0.20), excluding 0. In addition, working in the high and low levels of the embedded, individuals and organizations match the indirect effect of difference value of 0.04, 95% confidence interval (0.04, 0.17), and do not contain 0, and a further sign of individual and organization matching under various levels in the transformational leadership and Cenozoic intermediary effect between knowledge staff turnover intention has significant differences.

**TABLE 8:** Direct effects and mediating effects on different levels of job embedding.

Job embeddedness	Effect value	BootSE standard error	BootLLCI floor	BootLLCI ceiling
2.65 (M-1 SD)	-0.07**	0.03	-0.14	-0.02
3.22 (M)	-0.10**	0.04	-0.17	-0.04
3.79 (M + 1 SD)	-0.12**	0.04	-0.20	-0.05

**Denotes *P* < 0.01.

**FIGURE 2:**
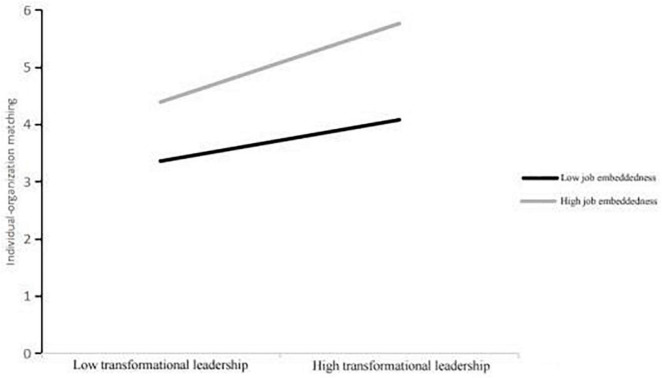
The moderating effect of job embedding on the relationship between transformational leadership and person–organization matching.

At the same time, exploring the relationship between work embeddedness and transformational leadership and individual–organizational matching is shown in Table 9. According to Table 9, the individual–organization matching score was plotted as a simple slope analysis graph (Figure 2) to further illustrate the interaction between transformational leadership and job embedding. The results showed that, at a high level of job embeddings, transformational leadership had a significant positive predictive effect on individual–organization matching (simple slope = 1.37, *P* < 0.01), and at a low level of job embeddings, transformational leadership still had a significant positive predictive effect on individual–organization matching (simple slope = 0.72, *P* < 0.01). *P* < 0.01, and the predictive effect increased with the mean increase of one standard deviation, indicating that the predictive effect of job embedment on individual–organization matching increased with the level of transformational leadership; H4, H5, and H6 were proved.

**TABLE 9:** The interaction between transformational leadership and job embedding.

	Coeff.	SE	*t*	*p*	LLCI	ULCI
Constant	3.3762	0.1023	32.9979	0.00**	3.1749	3.5774
Transformational leadership	0.3393	0.0486	6.9778	0.00**	0.2437	0.435
Job embeddedness	0.4894	0.0595	8.2313	0.00**	0.3724	0.6064
Int_1	0.1429	0.0524	2.7245	0.0068**	0.0397	0.246

**Denotes *P* < 0.01.

## Conclusion

### Research conclusion

This research draws the following conclusions: (1) transformational leadership negatively predicts the turnover intention of the new generation of knowledge workers. A wide variety of extant theoretical and empirical studies proposed that the behavior and values of transformational leadership play a crucial role in the development direction of employees, such as the innovation performance and turnover intention of employees (Gerlach et al., 2020). However, there are various types of employees, and these studies do not carry out in-depth research on a specific type of employee, such as the new generation of knowledge workers. Our results reveal that there is a complex relationship between transformational leadership behavior and the turnover intention of the new generation of knowledge workers. When leaders do not have convincing character and superb business ability, they cannot provide clear goal orientation and do not pay attention to material care and interpersonal care to create an organizational atmosphere, so the new generation of knowledge workers will have the idea of leaving. It shows that the willingness to stay off the new generation of knowledge workers not only depends on personal factors such as industry preferences but also pays attention to whether the leader’s behavior and values match with it. These findings provide theoretical insights into the dynamic development of the new generation of knowledge workers. (2) This study proves that individual–organization matching plays a partial mediating role between transformational leadership and the turnover intention of the new generation of knowledge employees, which has a certain contribution to the theoretical research on the relationship between transformational leadership and the turnover intention of the new generation of knowledge employees. Previous research provides circumstantial evidence for our inference; such as, Sok et al. (2021) think that it is only when the fit between FLEs, their roles, and the work climate is right that firms will get the best out of their employees. This research found that individual–organization fit acts as a partial mediator between transformational leadership and the turnover intention of the new generation of knowledge workers. It shows that transformational leaders can not only directly and negatively predict the turnover intention of the new generation of knowledgeable workers, but also negatively predict their turnover intention through the degree of matching between the new generation of knowledgeable workers and all aspects of the organization. These findings enrich existing theories and contribute to a better understanding of how transformational leadership influences the turnover intentions of the new generation of knowledge workers. (3) Job embeddedness has a moderating effect between transformational leadership and individual–organizational fit, and job embeddedness can positively moderate the mediating effect of personal–organizational fit between transformational leadership and the new generation of knowledgeable workers. Previous studies noted that job embedding has an important impact on employees’ job performance (Qian et al., 2022) and voluntary admission (Peltokorpi et al., 2022); such as, Ali et al. (2022) investigated the relationship between psychological capital and employee performance through the mediating role of job embeddedness. The research results showed that job embeddedness significantly mediates the link between psychological capital and employee performance. Chen (2022) believes that job embeddedness is positively correlated with employees’ innovative service behavior. Through job embeddedness, employees’ enthusiasm can be stimulated, thus promoting their innovation. In this research, by exploring the moderating effect of job embeddedness, transformational leadership can positively influence individual–organization fit at both high and low levels of job embeddedness, but when compared with low job embeddedness, transformational leadership can more positively affect the new generation of knowledgeable workers and organizations when job embeddedness is high. The matching of values, supply and demand, ability, and requirements makes it easier to retain a new generation of knowledgeable workers. Therefore, job embeddedness is mainly responsible for regulating the first half of the mediating path and has a positive regulatory effect. This provides more insight into which behaviors of transformational leaders can positively impact the new generation of knowledge workers.

### Management implications

Based on the research conclusions, this study draws the following management implications.

First, focus on cultivating the transformational leadership style of managers to enhance the retention tendency of the new generation of knowledgeable workers. Extant research suggested that transformational leadership style has a significant negative correlation with employees’ turnover intention, and employee performance mediates the connection between turnover intentions and TL. In other words, transformational leaders encourage employee performance, which, in turn, decreases their turnover intentions (Yücel, 2021). However, our research suggests that transformational leadership may not always use employee performance to influence employee turnover intention, especially for the new generation of knowledge workers. According to the conclusion of this study, transformational leadership can negatively predict the turnover intention of the new generation of knowledgeable workers, which means that leaders can weaken the turnover intention of this group by improving their own good morals, vision planning ability, personal charm, and interpersonal care. In terms of morality, leaders should not only set an example in their daily work but also pay attention to the influence of personal reputation and reputation on the willingness of the new generation of knowledgeable workers to follow when dealing with personal affairs; combined with the personal goals of the new generation of knowledgeable workers, leaders who regard the development of the new generation of knowledgeable workers as equally important as organizational development will inevitably lead to a high degree of organizational support perception among the new generation of knowledgeable workers and stimulate their sense of ownership to reduce their willingness to leave; in terms of charisma, the new generation of knowledgeable workers who are attracted by new things and are interested in unique personalities is often attracted by a certain superb ability of the leader. Therefore, leaders should always maintain a state of learning and improvement, constantly improve their business ability and ability to deal with interpersonal affairs, and show this mature and professional leadership quality to the new generation of knowledgeable workers to positively influence their loyalty and following. In terms of individual care, leaders can give corresponding help and support according to the career development status and family living conditions of the new generation of knowledgeable employees, such as helping the new-generation knowledgeable employees who have just entered society to formulate personalized career plans and reserve positions for the new generation of knowledgeable workers who need further education. This personalized care will satisfy the “self-awareness” of the new generation of knowledgeable workers to a certain extent and make them feel the sincerity of their leaders, thereby enhancing their trust and dependence on the organization.

Second, pay attention to the changes in the value, supply–demand, and ability–demand of the new generation of knowledgeable workers and the degree of matching with the organization, and strengthen the mediating role of individual–organization matching. Extant research has suggested that occupational stress significantly affects employees’ turnover intention (Cuc et al., 2022). However, our research shows that there is a certain correlation between employee turnover intention and organizational matching degree. The new generation of knowledgeable workers will gradually form a set of values, ways of thinking, and ways of doing things that fit the collective in the process of running in with the organization, and these concepts and behaviors are not static. In the process of adapting to the organization, due to the fierce social competition and peer pressure, the new generation of knowledgeable workers is susceptible to the interference of external noise and the temptation of petty profits, resulting in blurred career goals and difficulty in deciding the future development direction. You can only jump back and forth between different industries frequently. If things go on like this, it will not only be unfavorable for the new generation of knowledgeable employees to accumulate work experience and social contacts, but also the team will not be able to recruit talents who meet the needs of the post. Therefore, leaders should always pay attention to the changes in the degree of matching between the new generation of knowledgeable workers and the organization and use their good characteristics to adjust the relationship between the new generation of knowledgeable workers and the organization in a timely manner. When the new generation of knowledgeable workers falls into a spiritual trap that is not conducive to the long-term development of organizations and individuals, such as “fast-food culture,” leaders can organize the human resources department and their immediate managers to help the new generation of knowledgeable workers establish positive social values. Recognize self-worth and the direction of development. When the new generation of knowledgeable workers has obtained certain material rewards and work achievements in the organization, the leaders cannot still maintain the organizational supply on the material surface. From the perspective of fairness theory, relying only on material rewards will increase the economic pressure on enterprises and lead to errors in subjective judgments by employees. Leaders should combine non-material methods such as decentralization and decentralization to meet the high-level needs and organizational status of the new generation of knowledgeable workers. Negativeness needs to be avoided. Emotions led him to leave. From the perspective of organizational development, when the business capabilities of the new generation of knowledgeable workers are gradually strictly required, leaders should face up to the changes in the group in the process of self-improvement and correctly recognize the learning ability and energy of the new generation of knowledgeable workers. It will be depleted due to physical fitness, work environment, and family environment. Therefore, leaders should help the new generation of knowledgeable workers improve their business level by carrying out support activities such as career planning and skills training.

Third, pay attention to work embedded in transformational leadership and personal fit. According to the conclusions of this study, work in a high level of embeddedness under the transformational leadership can more actively influence personal organization match and, then, by raising the level of work embedded in Cenozoic knowledgeable employees can help to more positively affect individual transformational leader. Based on the study of talent competition strategies in the first- and second-tier cities, it is found that policies such as employment and living allowance for talents’ immediate family can meet the needs and expectations of the new generation of knowledgeable workers for community life. In addition, many listed companies tend to bestow conditional stock options to meet the economic needs of the new generation of knowledgeable employees. This kind of bundled income within the organization determines how long the employee can enjoy the dividend after staying in the post and also stipulates that the income from the stock options cannot be continued to enjoy bundled income after leaving the post. Based on the local economic environment and enterprise strength, after understanding the different needs of the new generation of knowledgeable workers in work and family, managers can exert the transformational leadership characteristics and play a moderating role of job embedding according to the development needs of the organization and the new generation of knowledgeable workers.

### Research deficiencies and prospects

Although this study provides some inspiration for related research, it also has the following shortcomings.

First, most of the respondents in this study are unmarried and around 25 years old, so they do not need to think too much about family issues such as children going to school for the time being. At the same time, in the process of data collection, this study did not investigate the family status of married respondents and failed to understand which community family factors had the most extensive influence on their turnover intention. In addition, in terms of the selection of moderating variables, this research mainly focuses on the analysis of job embeddedness to explore the impact of job embeddedness on the relationship between transformational leadership and the new generation of knowledge workers. However, the relationship between transformational leadership and the new generation of knowledge workers is very complex, and other factors may have a moderating effect on the relationship between transformational leadership and the new generation of knowledge workers, such as gender difference. Therefore, this study has certain limitations in exploring the role of community family factors in the relationship between transformational leadership and individual–organization fit. Therefore, in future research, the marital status of the respondents can be listed as one of the control variables, and gender can be used as the moderating variable between transformational leadership and individual–organizational matching, combined with the interview method, to explore whether community family factors will affect the relationship between transformational leadership and individual–organizational matching due to different marital status and whether gender differences will affect the relationship between transformational leadership and individual–organizational matching.

Second, the research samples in this study are mainly from Guangxi. Considering the differences between the north and the south and the potential characteristics of the autonomous region in future research, provinces with differences in economy and culture from Guangxi can be selected for research, and the sample size of the questionnaire will be increased to avoid regional bias. At the same time, it can also be divided and compared according to the nature of the work, the content of the work, etc., to explore whether variables such as transformational leadership can still negatively affect the turnover intention of the new generation of knowledgeable workers under different work content and working conditions. For example, programmers and other professional and technical new-generation knowledgeable workers are suitable for long-term home office due to job content and work equipment, so whether transformational leadership and job-embedded community factors have an impact on their turnover intention.

Third, the hypothesis section of this study focuses on the relationship between transformational leadership and the new generation of knowledge workers. However, work environment and work pressure can also be important factors. Therefore, future research will attempt to add factors such as work environment and work pressure to the analysis of the impact of transformational leadership on individual–organizational matching and the impact of individual–organizational matching on the turnover intentions of the new generation of knowledge workers.

## Data availability statement

The raw data supporting the conclusions of this article will be made available by the authors, without undue reservation.

## Ethics statement

This study was approved by the Ethical Committee of Guangxi University. We introduced our research purpose, goals, and plans to each employee and asked their permission to participate in this research. Employees could withdraw from the study at any time without penalization. We obtained written informed consent from all participants before data collection.

## Author contributions

BX and XW: conceptualization, methodology, software, validation, formal analysis, resources, writing—original draft preparation, writing—review and editing, visualization, supervision, project administration, and funding acquisition. QS: data management, validation, article arrangement, and formatting. All authors contributed to the article and approved the submitted version.
